# Mitochondrial recoupling: a novel therapeutic strategy for cancer?

**DOI:** 10.1038/bjc.2011.245

**Published:** 2011-06-28

**Authors:** G Baffy, Z Derdak, S C Robson

**Affiliations:** 1 Department of Medicine, VA Boston Healthcare System and Brigham and Women's Hospital, Harvard Medical School, 150 S Huntington Avenue, Room A6-46, Boston, MA 02130, USA; 2 Liver Research Center, Department of Medicine, Rhode Island Hospital and Alpert School of Medicine, Brown University, Providence, RI 02903, USA; 3 Liver Clinic, Department of Medicine, Beth Israel Deaconess Medical Center, Harvard Medical School, Boston, MA 02215, USA

**Keywords:** uncoupling proteins, UCP2, aerobic glycolysis, metabolic reprogramming, oxidative stress, p53

## Abstract

Recent findings link metabolic transformation of cancer cells to aberrant functions of mitochondrial uncoupling proteins (UCPs). By inducing proton leak, UCPs interfere with mitochondrial synthesis of adenosine 5′-triphosphate, which is also a key determinant of glycolytic pathways. In addition, UCP suppress the generation of superoxide, a byproduct of mitochondrial electron transport and a major source of oxidative stress. The near ubiquitous UCP2 becomes highly abundant in some cancers and may advance metabolic reprogramming, further disrupt tumour suppression, and promote chemoresistance. Here we review current evidence to suggest that inhibition of mitochondrial uncoupling may eliminate these responses and reveal novel anti-cancer strategies.

Cancer cells are exposed to seemingly adverse conditions such as hypoxia, nutrient limitation and immune defence mechanisms. Those surviving adaptive cancer cells have successfully responded to the selection pressure of the host microenvironment by subversive molecular changes that impact mitochondrial functions and promote glycolysis. These changes foster metabolic flexibility, autonomous growth, abrogation of programmed cell death, sustained angiogenesis and immune evasion (Hanahan and Weinberg, 2000). In clinical practise, these adaptive cellular responses might also manifest as chemoresistance. A better understanding of the molecular mechanisms that facilitate cancer cell survival should help guide novel therapeutic strategies.

## Mitochondrial homeostasis and uncoupling proteins (UCPs)

In normal cells, mitochondria integrate molecular pathways of energy production and biosynthesis, maintain redox balance, regulate intracellular calcium signalling and participate in cell fate decisions, including the initiation and execution of apoptosis. Mitochondria also has critical roles in the survival strategy of cancer cells (Frezza and Gottlieb, 2009).

**Figure 1:**
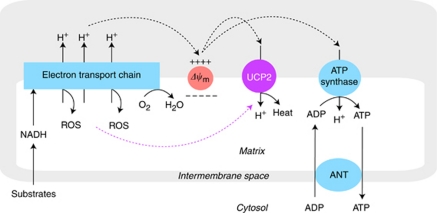
Oxidative phosphorylation and mitochondrial uncoupling. Substrate-derived electrons from glucose and fatty acid metabolism flow through complexes I–IV of the electron transport chain embedded in the mitochondrial inner membrane and the energy of this process is used for pumping protons (H^+^) from the matrix into the intermembrane space. The resulting proton gradient sustains the mitochondrial membrane potential (Δ*ψ*_m_), which drives ATP synthase (oxidative phosphorylation). The ATP and ADP are exchanged between the matrix and cytoplasm via ANT. Proton conductance (proton leak) induced by uncoupling proteins (exemplified here by the ubiquitous UCP2) competes for the same proton gradient, resulting in lower values of (Δ*ψ*_m_) and diminished production of ATP. Decrease in Δ*ψ*_m_ accelerates electron transport and mitochondrial respiration, limiting the odds for electron escape and production of superoxide, a prototype of ROS. Activation of UCP2 by ROS (purple dotted arrow) provides an important negative feedback mechanism for the regulation of Δ*ψ*_m_ and mitochondrial oxidant production. The colour reproduction of this figure is available at the *British Journal of Cancer* online.

Within mitochondria, the machinery of oxidative phosphorylation carries out high-yield adenosine 5′-triphosphate (ATP) synthesis at the expense of generating reactive oxygen species (ROS; Figure 1). Reducing equivalents generated by the tricarboxylic acid (TCA) cycle or by *β*-oxidation of fatty acids provide the electrons that are transported along the electron transfer complexes I–IV of the inner mitochondrial membrane (Nicholls and Ferguson, 1992). The energy of this process is coupled with outward translocation of protons across the inner mitochondrial membrane, defined as the mitochondrial membrane potential (Δ*ψ*_m_). Re-entry of protons to the mitochondrial matrix drives the ATP synthase (complex V) that converts adenosine 5′-diphosphate (ADP) to ATP. To complete the process, adenine nucleotide translocase (ANT) exchanges ADP for ATP across the mitochondrial inner membrane (Nicholls and Ferguson, 1992).

The mitochondrial electron transport chain (ETC) is an inherent source of intracellular ROS (Turrens, 2003). Although transported electrons are destined to reach molecular oxygen at the level of cytochrome oxidase (complex IV), some electrons escape the ETC at earlier steps and form superoxide, a major ROS variant, by single electron reduction of molecular oxygen (Brand *et al*, 2004). The levels of superoxide generation are high if the electron flow becomes sluggish and the half-life of mobile electron carriers is prolonged. This may occur when there is a supply/capacity imbalance of proton movements either due to accelerated metabolic rates (increased supply) or due to partial impairment of the mitochondrial respiratory complexes including the ATP synthase (decreased capacity; Skulachev, 1998).

As superoxide production is very sensitive to changes in Δ*ψ*_m_, mitochondrial ROS levels can be effectively controlled by the rate of proton re-entry (Brand, 1990). A considerable amount of protons may bypass the ATP synthase pathway and leak back to the mitochondrial matrix. This seemingly wasteful dissipation of the proton-motive force as heat energy is termed mitochondrial uncoupling (Brand, 1990). More important, this event is mediated by UCPs and represents the first line of antioxidant defence aimed at resolving mismatched outward and inward proton fluxes (Skulachev, 1998; Brand and Esteves, 2005).

The UCPs belong to the mitochondrial anion transporter superfamily located in the inner mitochondrial membrane (Boss *et al*, 1999). The UCP1 is the longest known UCP, confined to brown adipose tissue where it is highly abundant and accounts for adaptive thermogenesis. The UCP2, a more recently identified member of the UCP family has gained attention, as it is essentially ubiquitous and has also been shown to mediate proton conductance (Krauss *et al*, 2005; Nubel and Ricquier, 2006). The UCP2 is much less abundant than UCP1 and has no apparent role in thermogenesis. Instead, UCP2 has been implicated in free radical scavenging relevant to diverse physiological and pathological processes, including obesity, neurodegenerative diseases, ageing and cancer (Nubel and Ricquier, 2006; Baffy, 2010). The antioxidant effect of UCP2 has been well documented in a variety of *in vitro* and *in vivo* experimental systems using UCP2 overexpression, genetic ablation and pharmacological inhibition (Arsenijevic *et al*, 2000; Collins *et al*, 2005; Derdak *et al*, 2008). Interestingly, UCP2-mediated proton leak requires activation by superoxide and lipid peroxidation derivatives such as 4-hydroxynonenal and other reactive alkenals (Echtay *et al*, 2002; Brand *et al*, 2004). Thus, UCP2 may be considered primarily as a sensor and suppressor of mitochondrial ROS, with increasing functional impact at increasing levels of oxidative stress.

**Figure 2:**
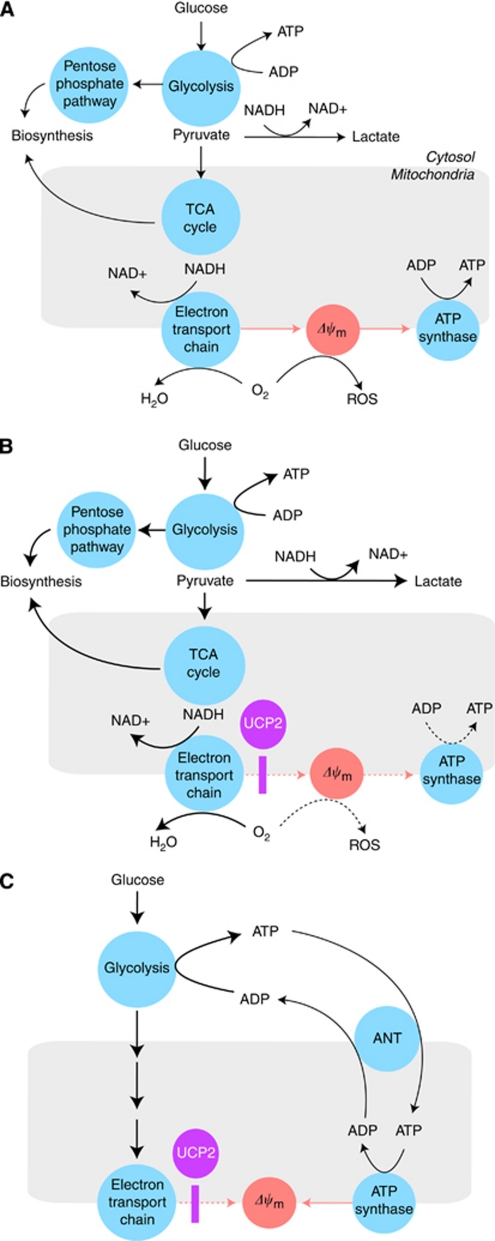
The UCP2 and energy metabolism. (**A**) In normal cells, catabolic and anabolic pathways intersecting in the mitochondrial TCA cycle are balanced by redox power. The NADH derived from substrate breakdown is primarily re-oxidised by mitochondrial respiration (electron transport chain). This process is coupled to ATP synthesis and depends on the magnitude of Δ*ψ*_m_ and availability of ADP. This may limit TCA flux and macromolecular biosynthesis rates. (**B**) In dysplastic or cancer cells, proton conductance induced by upregulated mitochondrial uncoupling (UCP2) lowers Δ*ψ*_m_ and not only disrupts both ATP synthesis and ROS generation (dotted arrows), but also dissociates the TCA cycle and upstream metabolic pathways from the constraints of oxidative phosphorylation. Under these conditions, the impact of glycolysis on bioenergetics and biosynthesis may increase without the burden of concurrently high mitochondrial ROS production (solid thick arrows). (**C**) Mitochondrial uncoupling may support biosynthesis in rapidly proliferating cells by an additional mechanism. High glycolytic rates in cancer cells may result in surplus ATP and feedback inhibition of glycolysis. This obstacle may be removed if ANT exports glycolytic ATP into the mitochondria where it is hydrolysed by ATP synthase. As reverse functioning ATP synthase pumps protons out of the matrix, ATP hydrolysis may sustain Δ*ψ*_m_ in mitochondria with impaired or futile (uncoupled) respiration. Therefore, UCP2 may create a mitochondrial ATP sink to boost glycolysis in cancer cells. According to this model, the sum of UCP2 and ANT effects may determine prevailing Δ*ψ*_m_ and account for any variability seen in cancer cells.

Although modulation of Δ*ψ*_m_ by inducible proton conductance is a prerequisite to UCP2-mediated control of ROS, lowering the proton-motive force by uncoupling has additional effects on cellular energy metabolism (Figure 2A). To sustain mitochondrial redox homeostasis, metabolite flux through the TCA cycle must be balanced with NADH re-oxidation rates by mitochondrial respiration. As the electron transport is coupled with proton translocation, biosynthetic and bioenergetic pathways are tightly linked and subject to regulatory constraints of mitochondrial respiration (Ainscow and Brand, 1999; Cortassa *et al*, 2009). Thus, UCP2 may prove pivotal in dissociating oxidative phosphorylation from other mitochondrial functions (Figure 2B). As it turns out, this dissociation is a key feature of metabolic and energetic transformation in cancer cells (DeBerardinis *et al*, 2008; Vander Heiden *et al*, 2009).

## UCP2 and metabolic reprogramming in cancer

In cancer cells, mitochondrial functions are modified to meet the special needs and liabilities of rapid and uncontrolled proliferation (DeBerardinis *et al*, 2008; Hsu and Sabatini, 2008). Perhaps the most prominent of these changes is the metabolic switch to aerobic glycolysis also known as the Warburg effect. Cancer cells increasingly favour glycolysis over mitochondrial oxidative phosphorylation as the source of ATP. This bioenergetic shift from mitochondria to the cytosol results in an increasingly aggressive cancer phenotype, indicating that aerobic glycolysis with generation of lactate is a successful adaptation strategy (Vander Heiden *et al*, 2009).

The molecular mechanisms underlying the Warburg effect and linking it to uncontrolled cell growth and proliferation are incompletely understood. There is mounting evidence to suggest cross-talk between changes in energy metabolism and oncogenic signalling pathways that collectively drive adaptive responses in cancer cells (Hsu and Sabatini, 2008; Vander Heiden *et al*, 2009). Several non-exclusive concepts have been proposed to explain the emergence of glycolytic phenotype in cancer. Enhanced glycolysis may allow high-rate ATP production with a selective advantage when competing for limited resources (Pfeiffer *et al*, 2001). These pathways feed into the pentose phosphate pathway to provide building blocks for nucleotide synthesis and NADPH for antioxidant defence; control the intrinsic apoptosis pathway via hexokinase-mediated inhibition of the voltage-dependent anion channel; and by producing excess lactate may sustain acidic microenvironments that are less habitable for normal cells, suppress immune responses and facilitate invasive growth (Gatenby and Gillies, 2004; DeBerardinis *et al*, 2008; Hsu and Sabatini, 2008).

One additional benefit of the Warburg effect is diversion of substrates from the ETC that may diminish the rate of mitochondrial ROS production (Brand and Hermfisse, 1997). Cancer cells often exhibit increased levels of intracellular ROS with complex and controversial biological effects (Burdon, 1995; Hussain *et al*, 2003). The ROS induce genomic instability and stimulate oncogenic pathways that promote cancer cell growth and survival. However, excessive and sustained ROS levels may lead to cell growth arrest, senescence and cell death by activating alternative signalling pathways and causing fatal macromolecular damage (Martindale and Holbrook, 2002; Hussain *et al*, 2003). Thus, effective regulation of intrinsic and treatment-induced oxidative stress is a critical ability of the surviving cancer cells that may acquire various forms of antioxidant defence (Martindale and Holbrook, 2002).

There is increasing evidence that UCP2 expression patterns are linked to cancer and may further modulate energy metabolism in response to high ROS levels (Baffy, 2010). Thus, UCP2 expression is increased in human colon cancer and may correlate with the degree of oxidative stress and neoplastic changes along with the ‘two-hit’ hypothesis and in the setting of adenoma–carcinoma transformation (Horimoto *et al*, 2004). The Warburg effect in certain leukaemia cells is linked to UCP2 activation (Samudio *et al*, 2008). Drug-resistant sub-lines of various cancer cells exhibit increased levels of UCP2, lower mitochondrial membrane potential and diminished susceptibility to cytotoxic effects (Harper *et al*, 2002). Overexpression of UCP2 in HepG2 human hepatoma cells limits oxidative stress and apoptosis in response to various challenges (Collins *et al*, 2005). Moreover, xenografts of UCP2-overexpressing HCT116 colon cancer cells retain growth in nude mice receiving chemotherapy, providing strong evidence that UCP2 upregulation is a plausible mechanism of chemoresistance in such studies (Derdak *et al*, 2008). These observations indicate that UCP2 is more than just a marker of increased ROS levels and serves as an important tool for reducing oxidative stress in adapting cancer cells.

What makes UCP2 an appealing molecular tool of adaptation for cancer cells? The evolutionary *raison d’être* of UCP2 seems difficult to comprehend, as increased inner membrane proton conductance not only allows efficient control of intracellular ROS, but it also disrupts oxidative phosphorylation. Importantly, ROS production is much more sensitive to uncoupling-mediated changes in Δ*ψ*_m_ than ATP synthesis (Miwa and Brand, 2003). Nonetheless, markedly enhanced UCP2 expression in non-transformed cells (primarily induced by fatty acids) may become a significant drawback as shown in pancreatic *β* cells of obesity-associated (type 2) diabetes. Here upregulated UCP2 leads to decreased ATP production and loss of glucose-stimulated insulin secretion (Zhang *et al*, 2001). Similarly, UCP2 abundance in hepatocytes is associated with limited ATP stores and energetic vulnerability of fatty liver (Chavin *et al*, 1999).

Curiously, the impact of UCP2 on cellular ATP production is not apparent in cancer cells that have a competitive growth advantage over normal differentiated cells (Derdak *et al*, 2008). Transformed cells may have substantial UCP2 upregulation seemingly without energetic compromise. This is predictable in cancer cells that exhibit high-rate ATP production by glycolysis, for as long as glucose remains available (Vander Heiden *et al*, 2009). Consumption of surplus ATP may in fact promote the Warburg effect in rapidly proliferating cancer cells by relieving allosteric inhibition of phosphofructokinase (PFK), a major enzyme controlling glycolysis (Israelsen and Vander Heiden, 2010).

A recently identified mechanism that indirectly consumes ATP and favors glycolysis is the heightened expression of endoplasmic reticulum ectonucleoside triphosphate diphosphohydrolase 5 in PTEN-null cells and following AKT induction (Fang *et al*, 2010). This organelle-associated UDPase promotes *N*-glycosylation of newly synthesised proteins and facilitates their correct folding in the endoplasmic reticulum by hydrolysing uridine 5′-diphosphate to uridine 5′-monophosphate (Israelsen and Vander Heiden, 2010). This activity is linked to ATP hydrolysis in the cytosol and has a positive effect on glycolytic rates (Fang *et al*, 2010). It is tempting to speculate that depletion of cytosolic ATP by UCP2-mediated uncoupling may similarly modulate PFK activity and thereby boost glucose metabolism in cancer.

Paradoxically, mitochondria may consume substantial amounts of glycolytic ATP to maintain critical homeostatic functions associated with Δ*ψ*_m_ if the proton-pumping activity of ETC becomes insufficient due to impaired respiration or in response to chemically induced uncoupling (Desquiret *et al*, 2006; Chevrollier *et al*, 2010). Under these conditions, the role of ATP synthase is reversed such that it contributes to Δ*ψ*_m_ by pumping out protons at the expense of ATP hydrolysis. To assist this process, cytosolic ATP is transferred to the matrix side by ANT2, an ANT isoform mainly expressed corresponding to the glycolytic activity in rapidly growing, undifferentiated cells (Chevrollier *et al*, 2010). Whether increased UCP2 expression helps cancer cells to transform mitochondria into a sink of glycolytic ATP by invoking the reverse function of ANT2 and ATP synthase remains to be seen. In addition, it is reasonable to speculate that reverse operating ANT, fuelled by glycolytic ATP, may provide a mechanism to counteract the effect of UCP2-mediated uncoupling. This might underpin the controversy about higher Δ*ψ*_m_ and impacts on cancer cells that are observed in different experimental systems (Figure 2C).

Evidence is gathering that inhibition of UCP2 may thwart metabolic adaptation and antioxidant defence mechanisms in cancer cells. Drug resistance is weakened by genipin in MX2 leukaemia cells that have abundant mitochondrial UCP2 (Mailloux *et al*, 2010). Genipin, an extract from *Gardenia jasminoides*, is a traditional Chinese remedy for type 2 diabetes that inhibits UCP2-mediated proton leak (Zhang *et al*, 2006). In MX2 cells, genipin decreases oligomycin-insensitive (uncoupling dependent) mitochondrial oxygen consumption and increases intracellular ROS levels in response to pro-oxidant agents such as menadione, doxorubicin and epirubicin (Mailloux *et al*, 2010). Similarly, genipin renders HT-29 and SW-620 human colon cancer cells more sensitive to cisplatin, as indicated by higher rates of mitochondrial ROS production and by decreased viability (Santandreu *et al*, 2010). These findings suggest that ROS toxicity induced by limiting inducible proton conductance via inhibition of UCP2 may improve responsiveness to conventional cancer drugs, identifying a potential novel approach to treat chemoresistance.

In keeping with the notion that partial breakdown of Δ*ψ*_m_ is the pivotal mechanism behind the antioxidant and anti-apoptotic effects of UCP2 in cancer cells, many of these effects are reproduced with the use of chemical uncouplers (protonophores). Protonophores are artificial compounds that allow protons to cross the lipid bilayer without the need for a channel or transporter. As recently reported, the protonophore carbonylcyanide *p*-trifluoromethoxyphenylhydrazone (FCCP) blocks the antitumour activity of various topoisomerase inhibitors and cisplatin in colon cancer cells (Derdak *et al*, 2008; Santandreu *et al*, 2010). Further studies will reveal to what extent various aspects of metabolic reprogramming in cancer cells can be modulated by artificial uncouplers.

## UCP2 and the p53 response in cancer

**Figure 3:**
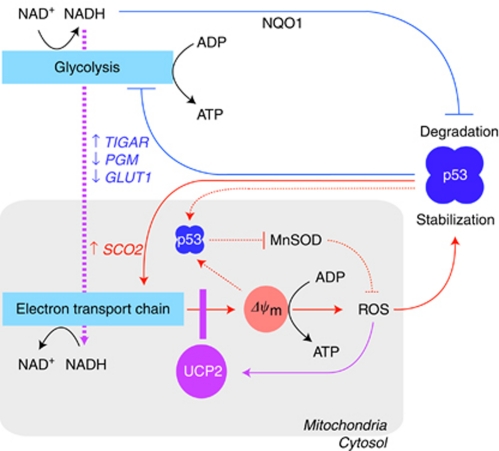
UCP2 and feedback regulation of p53. The tumour suppressor p53 controls metabolic pathways through multiple mechanisms. Transcriptional activation of SCO2 augments the capacity of mitochondrial electron transport, which is a major source of ROS (solid red lines). In addition, a fraction of p53 translocates to the mitochondrial matrix and directly inhibits MnSOD, further increasing ROS (dotted red lines). Metabolic stress resulting from these events promotes p53 stabilisation. Thus, pro-oxidant effects of p53 are regulated by multiple feed-forward amplification loops. The UCP2 is also activated by ROS (solid purple line) and may block these p53 responses by modulating the mitochondrial membrane potential (Δ*ψ*_m_) and breaking ROS-mediated p53 activation as well as interfering with p53 translocation to the matrix. In a negative regulatory loop, differential targeting of *TIGAR*, *PGM* and *GLUT1* genes by p53 may diminish glycolytic flux and NADH-mediated binding to NQO1, which would otherwise protect p53 from ubiquitin-independent degradation (solid blue lines). Because of higher mitochondrial respiratory rates, UCP2 may increase NADH shuttling to the mitochondria (dotted purple line), attenuate NQO1 activity and promote p53 degradation. The colour reproduction of this figure is available at the *British Journal of Cancer* online.

The impact of UCP2 on mitochondrial homeostasis is potentially so pervasive that it is difficult to identify a specific molecule or mechanism as the downstream effector of altered uncoupling in cancer cells. However, there is increasing evidence that p53, the main guardian of genomic integrity is a functional target of UCP2 (Figure 3). Modulation of mitochondrial ROS production by UCP2 may drive this interaction since stabilisation and activation of p53 is responsive to intracellular ROS (Lavin and Gueven, 2006). Depending on the level of oxidative stress, p53 may activate antioxidant or pro-oxidant mechanisms to allow cell growth arrest and damage repair or to initiate cell death pathways (Sablina *et al*, 2005). This dichotomy of p53-mediated cell fate decisions reflects the Janus-like pleiotropy of ROS biology. Although excessive accumulation of ROS may create a positive feedback loop for p53 and shift tumour suppressor mechanisms from repair to demise (Liu *et al*, 2008), UCP2 is likely to alter the spectrum of p53 responses by modulating ROS balance and assist the survival of cancer cells.

Several lines of evidence corroborate an opposing relationship between mitochondrial uncoupling and p53. First, UCP2 overexpression and diminished ROS levels in colon cancer cells interfere with post-translational phosphorylation of p53 by stress-activated protein kinases at the critical Ser^15^, Ser^33^ and Ser^46^ residues of its NH2 transactivation domain (Derdak *et al*, 2008). Second, p53 favors oxidative phosphorylation over glycolysis by regulating the transcription of several target genes, including synthesis of cytochrome oxidase 2, Tp53-induced glycolysis and apoptosis regulator, phosphoglycerate mutase and glucose transporter 1 (Vousden and Ryan, 2009), whereas UCP2 has a contrasting effect on cellular energy metabolism (Samudio *et al*, 2008). Thus, UCP2-overexpressing cancer cells increasingly display the Warburg effect (Derdak *et al*, 2008), and siRNA-mediated UCP2 knockdown leads to reversal of the glycolytic phenotype (Samudio *et al*, 2008). Third, translocation of p53 to mitochondria, an important step in the intrinsic apoptotic pathway, is blocked by the uncoupling action of FCCP in JB6 skin cells, whereas UCP2 knockdown promotes p53 translocation (Wang *et al*, 2010). This latter finding indicates that uncoupling may modulate mitochondrial protein trafficking in agreement with the notion that Δ*ψ*_m_ is a key determinant of the efficiency and rate by which nuclear-encoded proteins reach their mitochondrial destination (Martin *et al*, 1991).

Recent reports suggest that modulation of ROS levels is not the only mechanism by which UCP2 may affect cancer biology. Cellular abundance of p53 is primarily regulated by its rapid degradation, in part via an ubiquitin-independent 20S proteasomal pathway, which rapidly proceeds unless prevented by NAD(P)H-dependent binding of p53 to NAD(P)H:quinone oxidoreductase 1, a cytoplasmic flavone-containing quinone reductase (Tsvetkov *et al*, 2009). Accordingly, low reducing power promotes p53 degradation, whereas high reducing power favors p53 stabilisation (Tolstonog and Deppert, 2010). As mitochondrial uncoupling stimulates the rate of electron transport and helps recycling cytosolic NADH into NAD+, UCP2 may promote a redox balance that favors p53 degradation. One may therefore speculate that 20S proteasomal degradation is yet another process of metabolic sensing by which UCP2 may oppose p53 responses and support cancer cell survival.

It is estimated that about 60% of all human cancers harbour gain-of-function (dominant negative) or loss-of-activity p53 mutations, whereas in the remaining cases the function of wild-type p53 is disrupted by additional mechanisms (Harris and Levine, 2005). On the basis of available data, we may assume that UCP2 contributes to the dysfunction of wild-type p53 and targeting mitochondrial uncoupling by UCP2 inhibition or by some other ways may help restore the functions of p53 unless this is wholly incapacitated by mutations. Further studies are necessary to determine whether UCP2-mediated changes in cancer cells have a measurable impact on mutated p53, which could provide additional targets for anti-cancer therapy.

## Perspectives

So far, mitochondrial uncoupling in malignancy has been the interest of a relatively small group of investigators. There should be cautious optimism about UCP2 entering centre stage and becoming a novel therapeutic target in cancer. Selective inhibition of UCP2 may cancel many benefits of metabolic reprogramming in cancer cells. However, a number of technical difficulties need to be resolved before a feasible strategy can be developed for controlling UCP2 in particular, and mitochondrial uncoupling in general. The UCP2 is a ubiquitous protein with low tissue abundance and sheltered cellular localisation (Brand and Esteves, 2005; Krauss *et al*, 2005). Loss of UCP2 function has been shown to result in dysfunction of normal cells, questioning the safety margin of inhibition without selective targeting (Arsenijevic *et al*, 2000; Nubel and Ricquier, 2006; Baffy, 2010). This may be cause for particular concern due to excessive activation of immune cells with considerable UCP2 abundance at baseline (Arsenijevic *et al*, 2000). Crossreactivity of putative inhibitors with other UCPs should also be considered. Development of mitochondria-targeted inhibitors has been further hindered by the fact that the crystal structure of UCP2 is not available. Genipin and derivatives hold promise, but the precise molecular mechanism of genipin-mediated UCP2 inhibition and potential side effects from the crosslinking activity of genipin remain to be elucidated (Zhang *et al*, 2006; Mailloux *et al*, 2010).

In an attempt to identify new and selective small-molecule inhibitors of mitochondrial uncoupling, a very recent report describes chromane derivatives that may act as a surrogate of the inhibitory purine nucleotides (e.g., ADP and guanosine 5′-diphosphate) that bind deep inside the *α*-helical bundle core of UCPs (Rial *et al*, 2011). Chromanes inhibit basal proton conductance of UCP1 and UCP2 and sensitise HT-29 colon cancer cells to commonly used chemotherapeutic agents such as cisplatin and doxorubicin (Rial *et al*, 2011).

Altered cellular metabolism wields critical adaptive power in cancer cells. Changes in bioenergetics and biosynthesis sustain loops of reinforcement with oncogenic signalling pathways, rendering selected clones of cancer cells increasingly difficult to destroy. The robust strategy of unremitting growth and proliferation defines major attributes of energy metabolism in cancer, whereas it allows heterogeneity and redundancy that support successful defection. The UCP2 appears to be centrally positioned in this scheme as a potential modulator of redox balance, ATP synthesis, oxidative stress, intracellular oxygen distribution, apoptosis and mitochondrial protein trafficking. Different cancer cells may exploit different facets of mitochondrial uncoupling. Now we have to resolve how, which, and when to recalibrate in order to reap the most therapeutic benefit.
